# Supplementary material for: Extracellular Vesicles Allow Epigenetic Mechanotransduction between Chondrocytes and Osteoblasts
Source: Int J Mol Sci. 2021 Dec 10;22(24):13282. doi: 10.3390/ijms222413282 (PMC8703680; doi:10.3390/ijms222413282)
Supplement: Supplementary file 1 [file ijms-22-13282-s001.zip › ijms-1484104 supplementary.pdf]

Supplementary Data

Supplementary Table S1: Antibody information

Antibody	Concentration	Supplier	Cat. No.	Species
Anti-β-actin	1:1000	Sigma Aldrich	A2066	mouse
Anti-COX2	1:1000	Abcam, UK	ab15191	rabbit
Anti-COL2	1:50	Abcam, UK	ab34712	rabbit
Anti-Alix	1:1000	Santa Cruz, USA	sc-53538	mouse
Anti-TSG101	1:1000	Santa Cruz, USA	sc-7964	mouse
Anti-CD81	1:1000	Santa Cruz, USA	sc-166029	mouse
Anti-rabbit	1:10000	Invitrogen, USA	G-21234	goat
Anti-mouse	1:10000	Invitrogen, USA	G-21040	goat

Supplementary Figure:

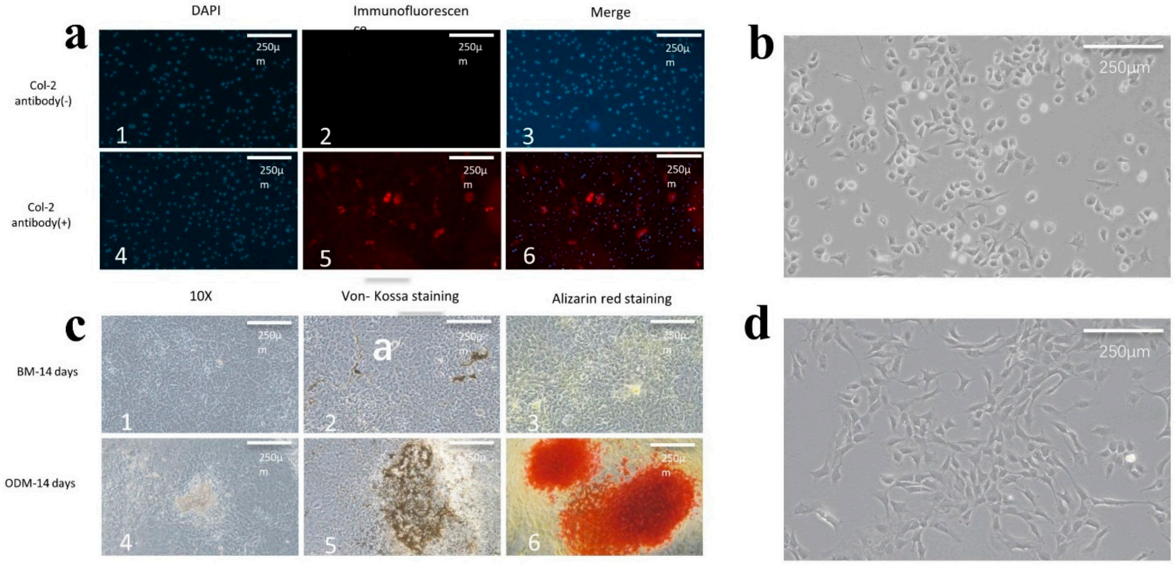

Supplementary Figure S1: Chondrocytes and osteoblasts were identified by histology and morphology methods. a. Col-2/collagen II Immunofluorescence with and without Col-2 antibody in chondrocytes. b. Typical chondrocytes from the articular cartilage of newborn rats looked like "cobblestone". c.Mineralized nodule formation of osteoblasts is illustrated by von Kossa staining and Alizarin Red staining after 14 days of culture in BM and OS. d. Osteoblasts from the calvarium of newborn rats showed irregular shapes such as triangle, fusiform, and polygon BM=Basal medium, ODM=Osteogenic differentiation medium.

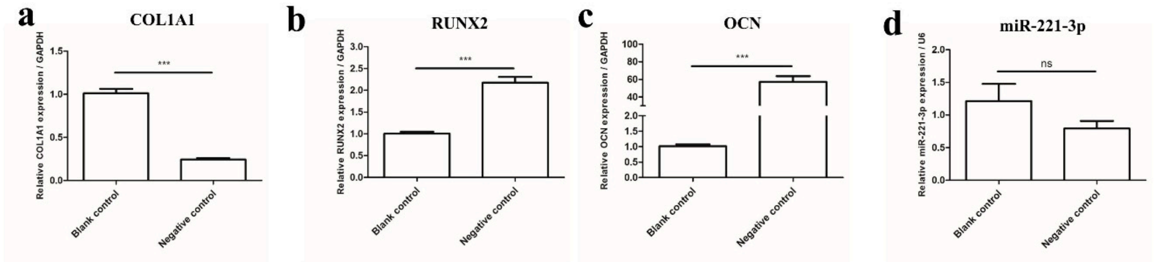

Supplementary Figure S2: Osteogenic markers and miR-221-3p expression were identified in osteoblasts. The expression level of osteogenic markers(a-c), COL1A1, RUNX2, and OCN and the expression(d) of miR-221-3p in osteoblasts cultured with ODM (Negative control group) and osteoblasts cultured with BM(blank control group). COL1A1, RUNX2, OCN relative to GAPDH, miR-221-3p relative to U6. ns stands for not statistically significant, ns stands for not statistically significant, $^{*}P<0.05$, $^{**}P<0.01$, and $^{***}P<0.001$ vs. corresponding control. BM=Basal medium, ODM=Osteogenic differentiation medium.

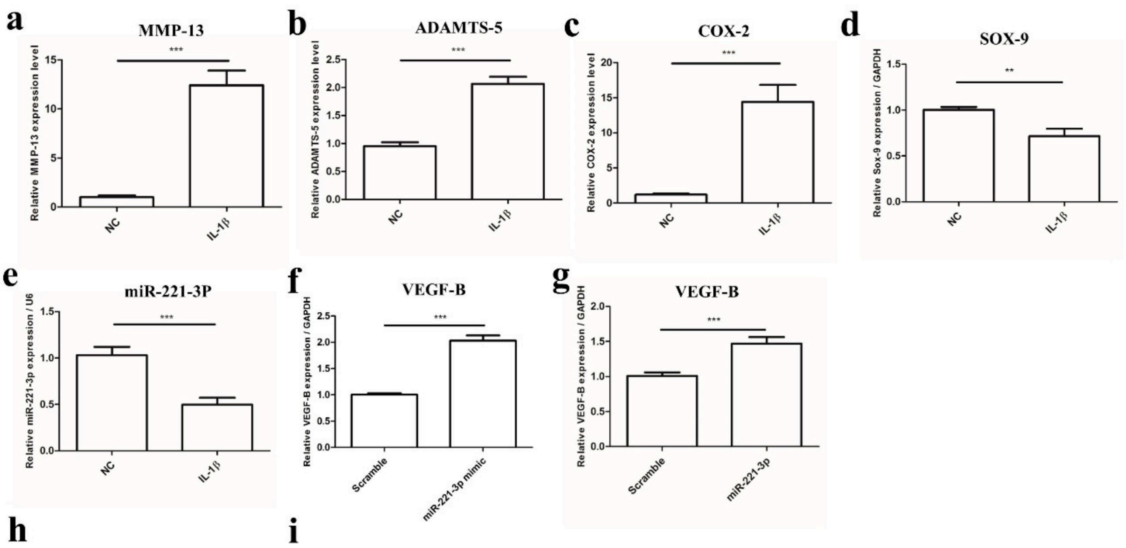

h

	U6	miR-221-3p
control-1	28.49	25.90
	28.03	26.14
	27.36	26.03
control-2	28.23	25.95
	28.30	26.04
	28.50	26.04
control-3	27.93	25.86
	28.22	25.78
	28.41	26.01

i

	U6	miR-221-3p
mimic-1	27.81	20.00
	27.41	19.84
	27.41	19.74
mimic-2	27.51	19.68
	27.54	19.81
	27.35	19.51
mimic-3	27.68	19.94
	27.66	20.03
	27.29	19.97

Supplementary Figure S3: a-d. The expression level of catabolic genes MMP-13(a), ADAMTS-5(b), COX2(c) in IL-1β-induced chondrocytes was significantly increased while the anabolic gene SOX-9(d) was decreased. e. The expression level miR-221-3p in IL-1β-induced chondrocytes was significantly decreased. f-g. After the transfection with miR-221-3p mimic and scrambled control in chondrocytes (f) and osteoblasts (g) respectively, the expression level of VEGF-B was significantly increased. h-j. The qRT-PCR analysis demonstrated that miR-221-3p was expressed in chondrocytes secreted EVs with a ~26 ct value(h) and overexpressed EVs secreted by transfected chondrocytes with a ~20 ct value (i). MMP-13, ADAMTS-5, COX2, SOX-9, VEGF-B relative to GAPDH, miR-221-3p relative to U6. **P<0.01 vs. corresponding control, ***P<0.001 vs. corresponding control.

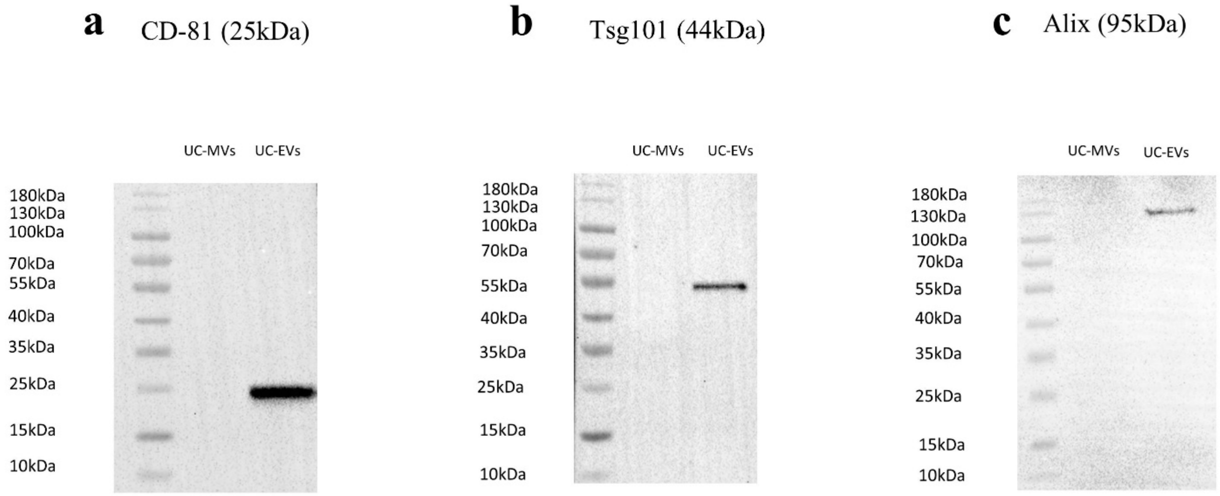

Supplementary Figure S4: Exosome-specific protein markers CD81 (a), TSG101 (b), and Alix (c) were detected by western blotting.

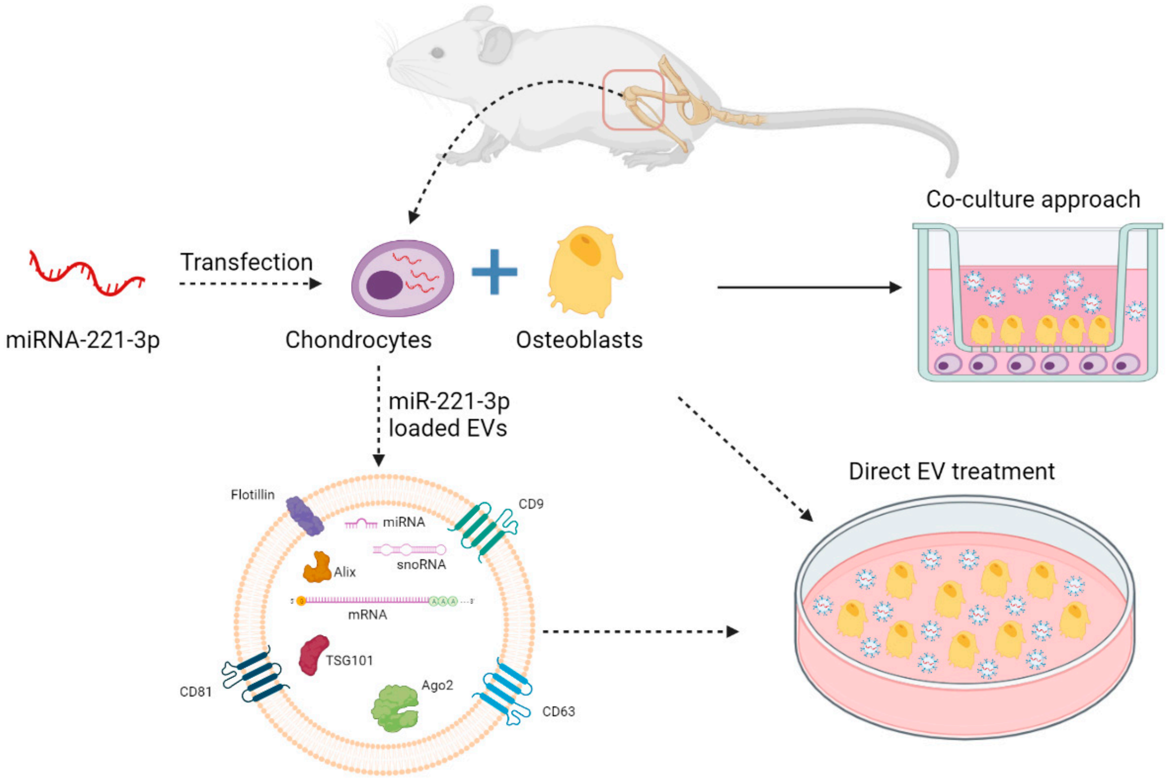

Supplementary Figure S5: Illustration of workflow. Chondrocytes and osteoblasts were isolated and identified from newborn rats. MiR-221-3p concentration in chondrocytes can be increased by the transfection method. In the next step, chondrocytes were cocultured with osteoblasts to study chondrocyte-osteoblast communication in the transwell. To understand the exact role of extracellular vesicles (EVs) in cell-cell communication, chondrogenic EVs were isolated and identified. Furthermore, osteoblasts were directly treated by miR-221-3p modified EVs derived from chondrocytes to study the communication of these two cell types. This figure was created with BioRender.com.
